# Heterotrimeric G-protein alpha-12 (Gα12) subunit promotes oral cancer metastasis

**DOI:** 10.18632/oncotarget.2437

**Published:** 2014-11-12

**Authors:** Chai Phei Gan, Vyomesh Patel, Constantinos M. Mikelis, Rosnah Binti Zain, Alfredo A. Molinolo, Mannil Thomas Abraham, Soo-Hwang Teo, Zainal Ariff Abdul Rahman, J. Silvio Gutkind, Sok Ching Cheong

**Affiliations:** ^1^ Oral Cancer Research Team, Cancer Research Initiatives Foundation (CARIF), Selangor, Malaysia; ^2^ Oral and Pharyngeal Cancer Branch, National Institutes of Dental and Craniofacial Research, National Institutes of Health, Bethesda, USA; ^3^ Department of Oro-Maxillofacial Surgical and Medical Sciences, Faculty of Dentistry, University of Malaya, Kuala Lumpur, Malaysia; ^4^ Oral Cancer Research and Coordinating Centre (OCRCC), University of Malaya, Kuala Lumpur, Malaysia; ^5^ Department of Oral and Maxillofacial Surgery, Tengku Ampuan Rahimah Hospital, Klang, Malaysia

**Keywords:** Oral squamous cell carcinoma, G-protein alpha-12, Lymph node, Metastasis

## Abstract

Oral squamous cell carcinoma (OSCC) has a propensity to spread to the cervical lymph nodes (LN). The presence of cervical LN metastases severely impacts patient survival, whereby the two-year survival for oral cancer patients with involved LN is ~30% compared to over 80% in patients with non-involved LN. Elucidation of key molecular mechanisms underlying OSCC metastasis may afford an opportunity to target specific genes, to prevent the spread of OSCC and to improve patient survival. In this study, we demonstrated that expression of the heterotrimeric G-protein alpha-12 (Gα12) is highly up-regulated in primary tumors and LN of OSCC patients, as assessed by quantitative polymerase chain reaction (qPCR) and immunohistochemistry (IHC). We also found that exogenous expression of the constitutively activated-form of Gα12 promoted cell migration and invasion in OSCC cell lines. Correspondingly, inhibition of Gα12 expression by shRNA consistently inhibited OSCC cell migration and invasion *in vitro*. Further, the inhibition of G12 signaling by regulator of G-protein signaling (RGS) inhibited Gα12-mediated RhoA activation, which in turn resulted in reduced LN metastases in a tongue-orthotopic xenograft mouse model of oral cancer. This study provides a rationale for future development and evaluation of drug candidates targeting Gα12-related pathways for metastasis prevention.

## INTRODUCTION

Cancer metastasis is a major hurdle for cancer treatment as it adversely impacts the quality of life and patient survival [1]. Oral squamous cell carcinoma (OSCC) predominantly spreads via the lymphatic route, and primarily to the cervical lymph nodes (LN). The presence of cervical LN metastases remains the most significant prognostic indicator in OSCC patients [2–4]. The two-year survival for OSCC patients with LN metastasis is ~30% compared to 80% for those without LN metastasis [5]. Therefore, the elucidation of key molecular mechanisms involving OSCC spread from their primary site affords an excellent opportunity to target these genes to prevent and stop the spread of OSCC and to improve patient survival.

Gα12 and Gα13 are members of the G12 subfamily of heterotrimeric G-proteins [6]. Activation of the G12 subfamily regulates a wide variety of important cellular events and responses that range from embryonic development [7–9], platelet activation [10, 11], angiogenesis [12], to apoptosis [13]. Most notably, however, of the two α-subunits, (Gα12 and Gα13), it is Gα12 that has consistently been demonstrated to play a key role in cancer development and progression, largely due to its potency in oncogenic transformation [14, 15].

There is now compelling evidence that Gα12 promotes cancer metastasis [16–19]. We have previously reported that Gα12 was significantly up-regulated in OSCC biopsies when compared to normal oral mucosa regardless of risk habits of the patients [20]. In this study, we sought to further investigate the functional impact of Gα12 up-regulation on OSCC development and progression. From our emerging data, we demonstrated that inhibiting G12 signaling results in reduced OSCC metastasis to LN, thereby identifying a potential therapeutic target to prevent the spread of OSCC and a range of other malignancies.

## RESULTS

### Gα12 is up-regulated in OSCC

**Figure 1:**
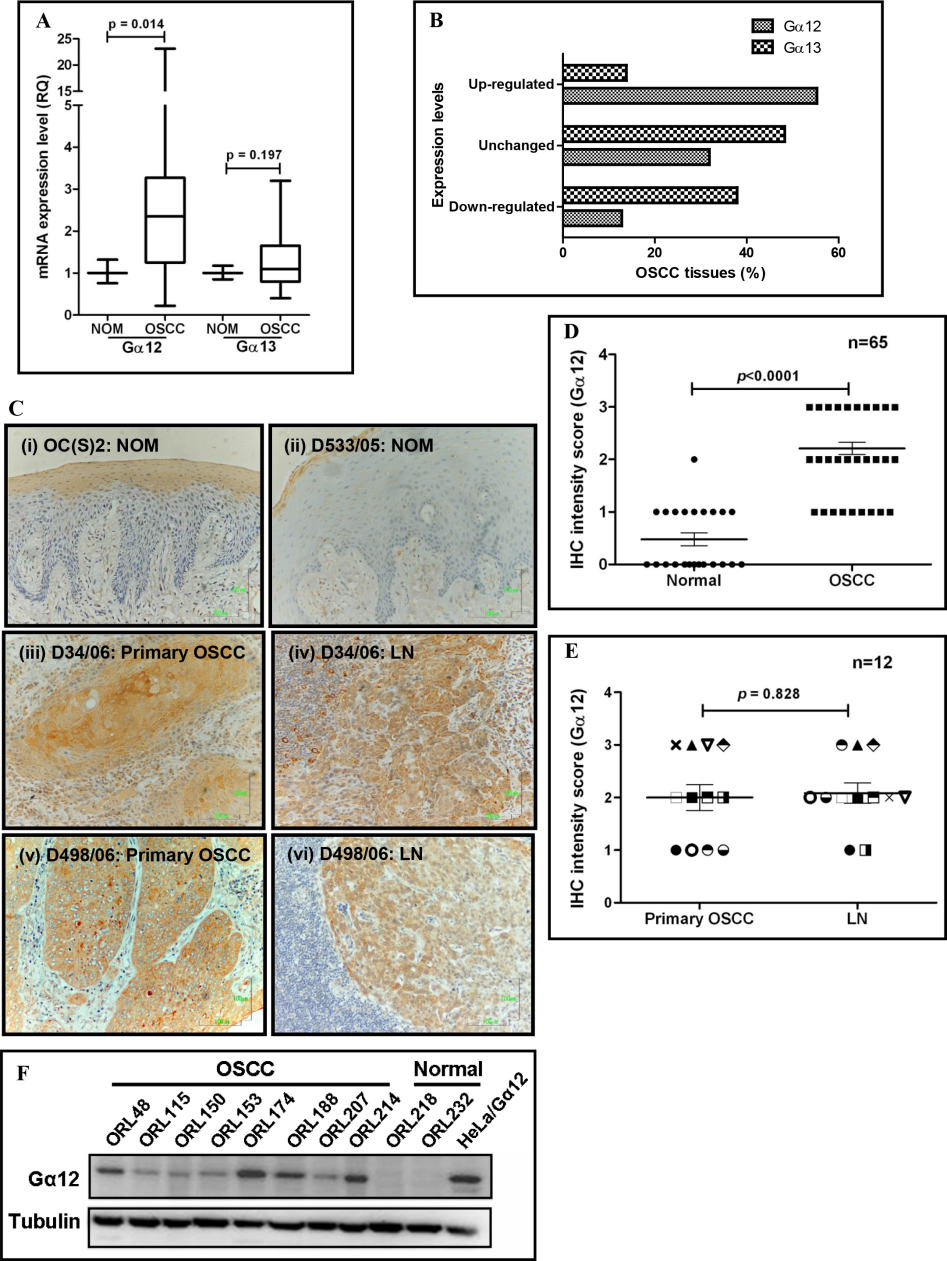
Overexpression of Gα12 in oral squamous cell carcinoma (OSCC) tissues and cell lines **(A)** qPCR analysis indicated that Gα12 was significantly overexpressed at the mRNA level in OSCC tissues. As indicated by the box and whiskers plot, OSCC tissues had a median of 2.35-fold increase in Gα12 expression, with a maximum level of 23-fold overexpression compared to normal oral mucosa (NOM). Meanwhile, the median of Gα13 expression is 1.1-fold, suggesting no differential expression between OSCC and NOM. **(B)** Gα12 mRNA levels were found to be overexpressed in 55.3% of OSCC tissues examined, and majority of the OSCC tissues had down-regulation of Gα13, when using a cut-off fold-change value of ≥ 2. **(C)** IHC images showing NOM tissues have negative staining for Gα12 (panel i & ii), but the primary tumors and the LN of matched cases showed overexpression of Gα12 (panel iii & iv; panel v & vi). Images were captured using 200x objective. **(D)** IHC staining analysis indicated that Gα12 is overexpressed in the primary tumors compared to NOM tissues. **(E)** Expression of Gα12 was also retained in the OSCC cells that metastasized to the lymph nodes (LN). Each symbol represents one patient. Error bars in IHC analyses represent standard error of the means (SEM) of Gα12 expression for the total number of tissues examined. **(F)** Western blot indicated Gα12 is overexpressed in all OSCC cell lines and absent in normal oral keratinocyte primary cultures. α-tubulin was used as an endogenous control and squamous non-oral HeLa cells transformed to overexpress Gα12 was used as a positive control.

Previously, we reported that Gα12 mRNA levels were up-regulated by more than two-fold in OSCC [20]. In this study, we sought to further validate these observations in an independent set of specimens and to assess if high levels of Gα12 were likely to have a biological impact on tumor progression. Firstly, as Gα12 share 67% sequence similarity with Gα13 [6], we examined mRNA levels of both molecules from the same tissue sample set. We confirmed that Gα12 mRNA levels were significantly up-regulated in OSCC (p=0.014), compared to Gα13 levels, which were essentially unchanged between OSCC and NOM tissues (Figure 1A). Furthermore, we found that the majority of OSCC tissues (26/47; 55.3%) had mRNA levels of Gα12 that were elevated ≥2 fold, further indicating the overexpression of Gα12 in this cancer type (Figure 1B). Consistently, IHC analysis demonstrated that the protein levels of Gα12 were overexpressed in OSCC tissues and tumor cells that have spread to the LN, but not in the NOM tissues (Figure 1C). In particular, Gα12 protein was significantly overexpressed in 34 of 43 (79.1%) OSCC cases and majority of these have the intensity score of 2 or 3 thus confirming our prior observations (Figure 1D). In contrast, 22 of 23 (95.7%) of NOM tissues showed low levels of Gα12 (intensity score 0–1). Although Gα12 was differentially expressed between OSCC and NOM, Gα12 was not associated with any of the clinical parameters, as detailed in Table 1. We next examined Gα12 protein levels in 12 LN positive tissues that were matched to the primary tumors from the same patient, and found that 10 of 12 patient samples (83.3%) had Gα12 overexpression in tumor cells that had metastasized to the cervical LN. Overall, Gα12 staining intensity was essentially similar between primary tumors and the matched LNs in majority of the cases (7/12), indicating that the high levels of Gα12 is retained in both primary tumors and LN metastasis, and 3/12 (25%) cases had increased Gα12 levels in the LN compared to the primary tumor (Figure 1E). However 1/12 showed consistently low expression of Gα12 in both the primary tumor and LN, while the remaining one case, showed high Gα12 levels in the primary but low in the matched LN. For potential detailed functional analysis of these observations, we next assessed the protein levels of Gα12 in a panel of OSCC cell lines previously established in our laboratory. From our data, we found that Gα12 was consistently overexpressed in the OSCC cell lines but not in normal oral keratinocytes primary cultures (ORL218 and ORL232; Figure 1F). In particular, 4 OSCC cell lines (ORL48, ORL174, ORL188 and ORL214) had the highest Gα12 expression with reference to the HeLa cells transduced to overexpress Gα12.

**Table 1:** Association of Gα12 protein expression with clinicopathological data

Characteristics	Gα12 expression			
	Patient n	Low n (%)	High n (%)	p-value
**Broder's grading**				
Well	16	3 (18.8)	13 (81.2)	0.709+
Moderate	20	5 (25.0)	15 (75.0)	
**Tumor size**				
T1 & T2	16	6 (37.5)	10 (62.5)	0.123+
T3 & T4	23	3 (13.0)	20 (87.0)	
**Lymph nodes**				
Negative	21	4 (19.0)	17 (81.0)	0.706+
Positive	18	5 (27.8)	13 (72.2)	
**Stage**				
I/II	8	2 (25.0)	6 (75.0)	1.000+
III/IV	31	7 (22.6)	24 (77.4)	
**Pattern of invasion**				
Cohesive	10	1 (10.0)	9 (90.0)	0.410+
Non-cohesive	31	8 (25.8)	23 (74.2)	

### Activation and expression of Gα12 promotes OSCC cell migration and invasion

**Figure 2:**
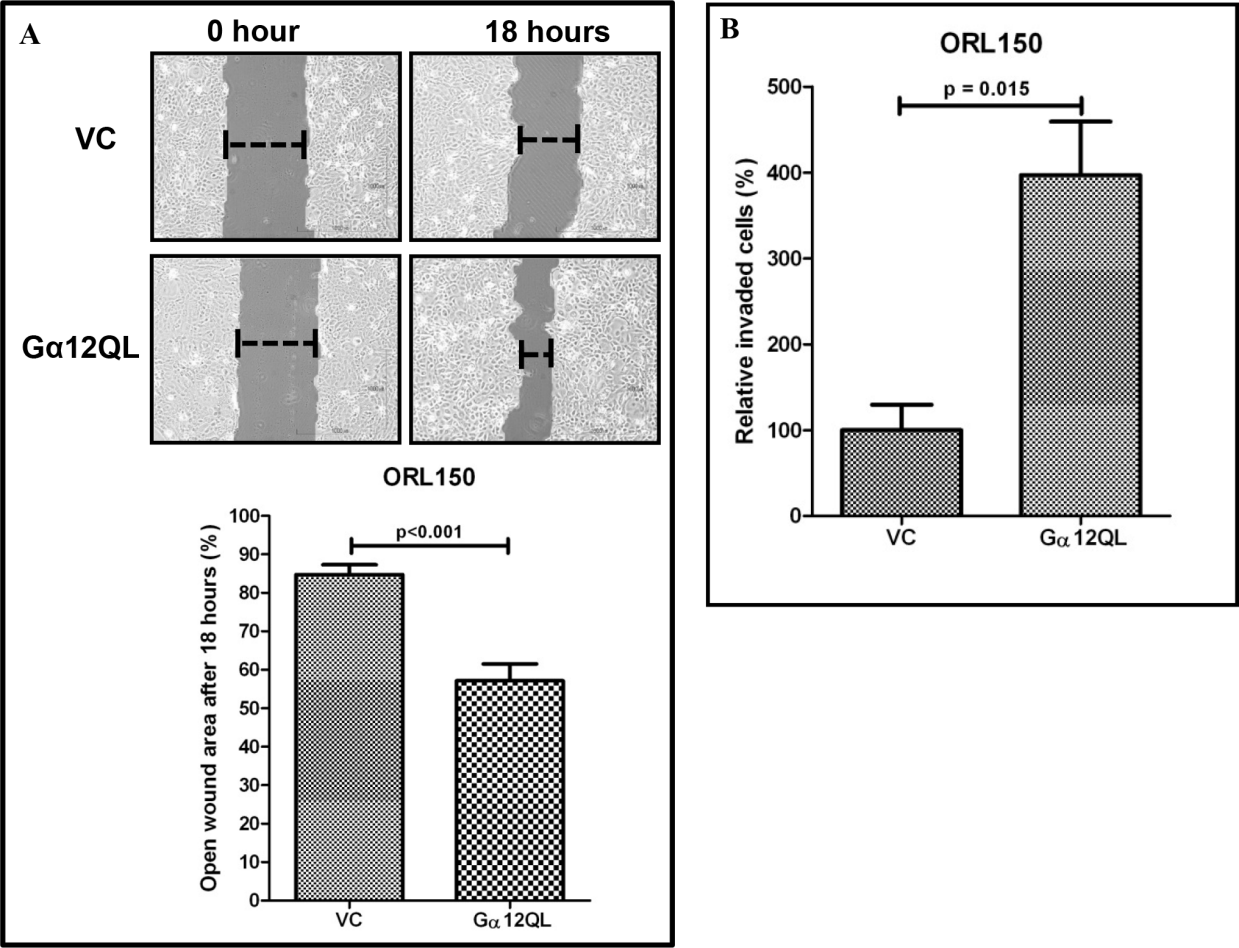
Activation of Gα12 promotes OSCC cell migration and invasion **(A)** The functional effect of the constitutive activated Gα12 (Gα12QL) on cell migration was assessed using scratch assay. Increased cell migration was observed in ORL150 cells that overexpress Gα12QL as indicated by a significant reduction in the open wound area, as compared to the ORL150/VC cells after 18 hours. **(B)** Similarly, ORL150/Gα12QL cells demonstrated 4-fold increase in invasion in comparison to the ORL150/VC cells. NIH3T3 conditioned medium was used as chemoattractant. The graphs are the representative results of 3 experimental repeats. Error bars represent SEM for the replicates tested in an experiment.

From our previous data, we chose ORL150 cells, largely due to the lower Gα12 levels amongst the OSCC cell lines examined, to ectopically overexpress the constitutively active form of Gα12 (Gα12QL) using pLXRN retrovirus transduction. Exogenous expression of Gα12QL in ORL150 stimulated Gα12 signaling, as indicated by the activation of down-stream RhoA (supplementary Figure 1). We next determined if this Gα12 activation was going to have an effect on cell migration. As seen in the scratch assay, we observed that the open wound area was 56.3% in ORL150/Gα12QL cells when compared to 85.7% in the vector control (VC) cells after 18 hours, indicating that expression of Gα12QL significantly increased the rate of migration of ORL150 cells in closing the open wound area (p<0.001; Figure 2A). Further, the transwell invasion assay demonstrated that ORL150/Gα12QL cells had a significant increase in the number of cells invading through the matrigel barrier in response to NIH3T3 cell-conditioned medium, compared to the VC cells (Figure 2B).

**Figure 3:**
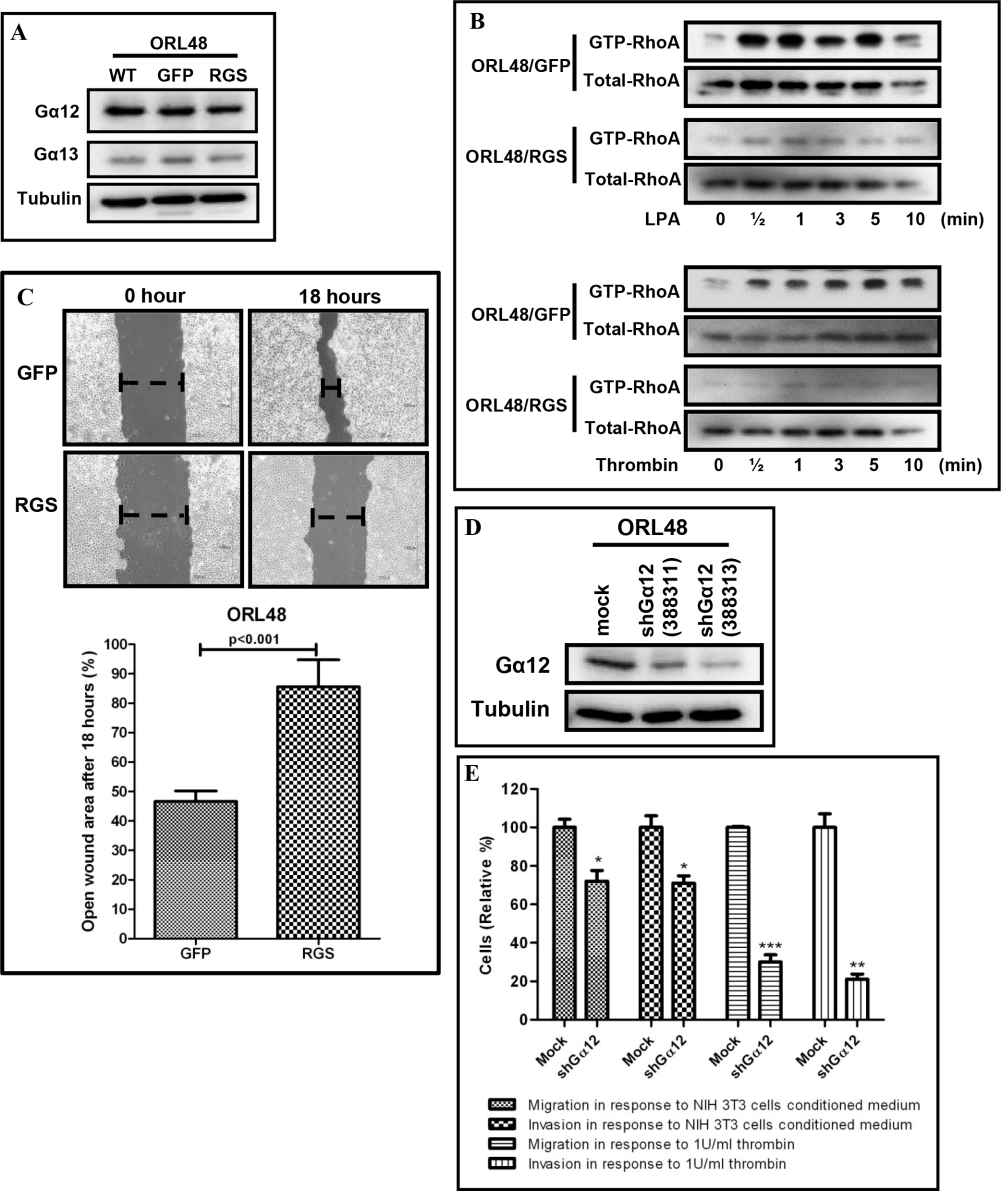
Inhibition of G12 signaling and knockdown of Gα12 in OSCC **(A)** Total protein levels of Gα12 and Gα13 were not affected in ORL48 cells transduced with GFP-fused RGS chimeric molecule. **(B)** Endogenous levels of activated RhoA were reduced in ORL48/RGS cells and remained inhibited even up to 10 minutes post LPA and thrombin stimulation, as compared to the ORL48/GFP control. **(C)** In scratch assays, the expression of GFP-fused RGS chimeric molecule in ORL48 consistently inhibited cell migration, as indicated by a significant larger open wound area compared to the ORL48/GFP control cells after 18 hours. **(D)** The expression of Gα12 in ORL48 which endogenously expresses high levels of Gα12 was knockdown using 2 separate shRNA sequences for Gα12 as shown by western blot. **(E)** Gα12 knockdown in ORL48 by shRNA inhibited OSCC cell migration and invasion in the transwell assays. ORL48/shGα12 cells showed a significant reduction in cell migration and invasion when tested with 2 different chemoattractant (NIH3T3 conditioned medium and 1U/ml thrombin). The graphs are the representative results of 3 experimental repeats. Error bars represent SEM for the replicates tested in an experiment.

Previous work has reported that both Gα12 and Gα13 of the G12 sub-family promote Rho activation by binding to the regulator of G-protein signaling (RGS) domain of RGS-containing Rho guanine nucleotide exchange factors (GEFs) [19, 21]. Here, we sought to inhibit both Gα12 and Gα13 signaling in OSCC by expressing a chimeric molecule consisting of GFP fused to the RGS domain of PDZ-RhoGEF in ORL48 cells (ORL48/RGS) (supplementary Figure 2). ORL48 cells were used for these studies due to high endogenous levels of Gα12 protein as well as the ability to form tumors when xenografted in mice. Expression of the chimeric molecule did not alter the endogenous levels of total Gα12 and Gα13 in ORL48 cells (Figure 3A), but was demonstrated to behave as a dominant negative mutant for G12 signaling by inhibiting LPA and thrombin dependent RhoA activation (Figure 3B). We observed that the endogenous level of activated RhoA was slightly higher in the ORL48/RGS control (0 time point). The levels of activated RhoA in ORL48/GFP control were markedly induced upon LPA and thrombin stimulations, respectively as early as 30 seconds. Interestingly, the levels of activated RhoA in ORL48/RGS remained low for up to 10 minutes of LPA and thrombin stimulations, indicating that the expression of the GFP-fused RGS chimeric molecule inhibited LPA- and thrombin-mediated G12-RhoA signaling axis.

As the presence of RGS inhibited the G12-mediated RhoA activation biochemically, we next examined if the presence of RGS in ORL48 was likely to confer a functional outcome. Here, we showed that the inhibition of G12 signaling by the GFP-fused RGS chimeric molecule caused a significant reduction of migration capacity as indicated by the open wound area in ORL48/RGS, which was 85.6% compared to 45.6% in the ORL48/GFP cells (p<0.001, Figure 3C). To confirm that this reduction in migration was due to reduced Gα12 functional activity through RGS, we decided to use a knockdown approach of reducing endogenously high levels of this protein in ORL48 cells. As seen in Figure 3D, the two shRNA sequences for Gα12 were able to reduce Gα12 protein levels to 44% (shGα12 322311) and 22% (shGα12 322313), and when these cell populations with controls were tested for their potential in cell migration and invasion, we noted that there was a significant reduction in cell motility and invasiveness in ORL48/shGα12 cells, compared to mocked transfected cells (Figure 3E). Knockdown of Gα12 in ORL48 led to ~30% and ~70% reduction in cell migration and invasion in response to the NIH3T3 cells conditioned medium and thrombin, respectively. Altogether, these findings strongly suggest that both activation as well as overexpression of Gα12 can lead to OSCC cell migration and invasion.

### Inhibition of Gα12 signaling reduced OSCC metastasis to LN in the orthotopic xenograft model

**Figure 4:**
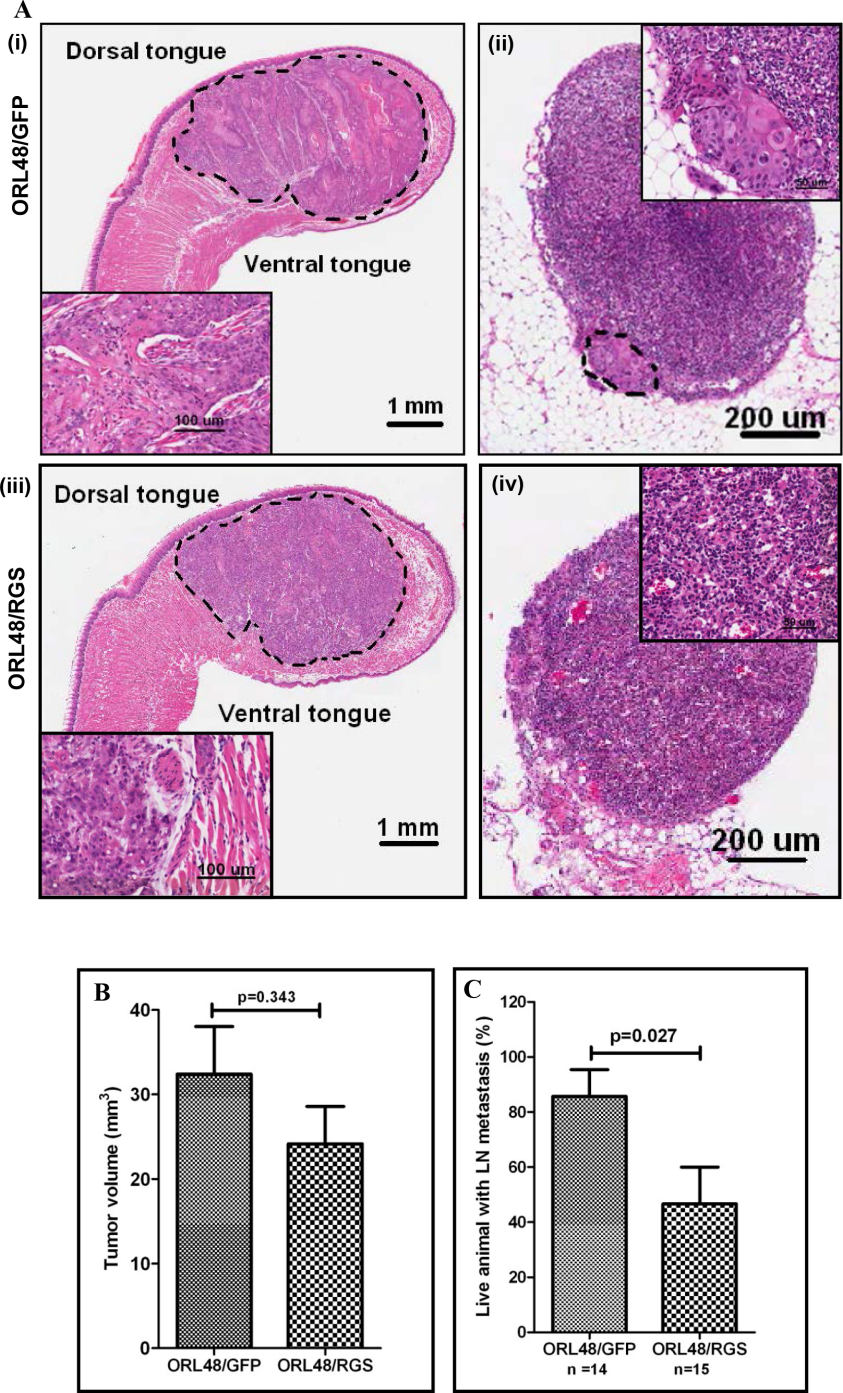
G12-RhoA signaling axis is involved in OSCC metastasis to cervical lymph nodes (LN) **(A)** H&E-stained tissue section of the orthotopically implanted OSCC cells into the tongue. ORL48/GFP control cells (i) and ORL48/RGS (iii) formed primary tumors growing into the anterior half of the tongue after day 20 post-implantation. Insets are higher magnification of the tumor area. Histologic evaluation of H&E-stained sections of the representative cervical lymph node showing the metastatic growth of ORL48/GFP control cells in the area rounded by dotted line. Inset is the higher magnification of the metastatic area (ii). Histological section of a non-invaded lymph node in the ORL48/RGS group (iv). **(B)** The growth of ORL48/GFP (control) primary tumors on the tongue were larger than those of the ORL48/RGS tumors, but the differences were not statistically significant. **(C)** Increased number of metastatic LN was found in mice carrying orthotopic ORL48/GFP control tumors, as compared to ORL48/RGS tumors. Animals that died of the disease before day 20 post-implantation were not included in the analysis as their LN were difficult to retrieve. The bar chart represents the average percentage of total animals that showed LN metastasis in 3 sets of experiments, while the error bars are SEM for these experiments.

OSCC frequently metastasize to the lymphatic basin in the neck area. Given that the oral tongue is composed of a dense lymphatic network, we developed an orthotopic model of OSCC metastasis by injecting ORL48 cells into the posterior of the tongue of NOD/SCID mice. Following the same model as previously reported [22], ORL48 cells formed moderately differentiated tumors within 20 days and were noted to be highly aggressive, invading the muscle and surrounding tissues (supplementary Figure 3A). More importantly, ORL48 cells also metastasized into the cervical LNs (supplementary 3B). With this information of the physiopathological behavior of ORL48 cells *in vivo*, and that the overexpression of RGS inhibits G12 signaling in ORL48 cells *in vitro*, we next assessed the impact of ORL48/RGS cells on OSCC spread to LN in the orthotopic-tongue mouse model. Both ORL48/GFP and ORL48/RGS cells formed ulcerating and highly aggressive tumors on the tongue of NOD/SCID mice after 20 days. Histopathological evaluation of the primary tumors indicated that the tumor had invaded into the muscle and the surrounding tissues (Figure 4A, i and iii). Perhaps of importance, no difference in the primary tumor volume was observed between the two groups (Figure 4B). Furthermore, we observed that 12 out of the 14 (85.7%) animals injected with ORL48/GFP control cells had OSCC spread to their cervical LN, while only 7 out of 15 (46.7%) animals injected with ORL48/RGS cells showed metastatic spread (Figure 4A, ii and iv). This difference in prevalence of LN metastasis (Figure 4C; p=0.027), suggests that inhibition of G12-RhoA signaling axis is not involved in regulating tumor growth but is pivotal for the inhibition of OSCC metastatic progression. In addition, we noticed that mice orthotopically injected with ORL48/GFP cells had higher mortality rate, as 5 out of 19 mice died before day 20 whilst no deaths were noted in the corresponding ORL48/RGS cohort during this experimental period (data not shown). Taken together, our results indicated that targeting the G12 signaling pathway may be an effective approach to prevent metastasis burden in OSCC patients.

### Gα12 is not involved in OSCC cell proliferation

**Figure 5:**
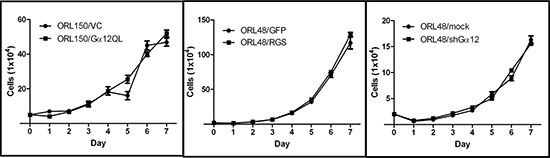
Gα12 is not involved in regulating OSCC proliferation No differences in cell growth were observed whether Gα12 was constitutively activated (Gα12QL) in ORL150 a cell line with low Gα12 expression or when Gα12 signalling was inhibited either by the GFP-fused RGS chimeric molecule (RGS) or by knockdown using shRNA (shGα12) in ORL48, a cell line with high levels of Gα12 expression. The graphs are the representative results of 3 experimental repeats. Error bars represent SEM for the replicates tested in an experiment.

To examine the effect of Gα12 on OSCC cell growth, we compared ORL150 cells expressing Gα12QL with VC cells. Activation of Gα12 had no effect on OSCC cell growth (Figure 5). This observation was confirmed in ORL48/RGS and ORL48/shGα12 cells, where no significant differences in the proliferation rate were found when compared to their respective control cells. These findings were consistent with our observation in the animal studies, whereby inhibition of Gα12 signaling has no effect on tumor volume (Figure 4B), suggesting that Gα12 is not involved in driving OSCC cell proliferation.

## DISCUSSION

When the spread of OSCC involves LN metastasis, the prognosis of the patient is significantly reduced and treatment becomes limited and invariably aggressive. Unfortunately, approximately half of OSCC patients present with metastatic cervical nodes at the time of diagnosis or follow-up [23], hence understanding the molecular mechanisms underlying OSCC progression from a localized growth to LN metastasis is critical to effectively prevent disease recurrence and ultimately improve survival. In this study, we further validated our previous observations that Gα12 is overexpressed in an independent OSCC sample cohort, suggesting an oncogenic role for Gα12 that warranted further investigation. Data from our current findings are in-line with studies reported in other solid cancers including nasopharyngeal cancer [18], adenocarcinomas of the breast and prostate [16, 17], as well as cell lines derived from different tumor origins [24]. The basis of the elevated Gα12 levels observed in our samples is unclear, but emerging data from our group have noted that all our OSCC cell lines with the exception of ORL214 showed chromosomal amplification at the region where Gα12 resides (7p22.2) (unpublished copy number alterations data). More importantly, studies have shown that Gα12 in the overexpressed and constitutively activated form harbors transforming capability [14, 15]. Studies on other Gα subunit family members showed that constitutively active mutants of Gαq and Gα11 are considered as driver oncogenes in 66% of ocular melanomas [25]. However, unlike the activating mutations found in other Gα subunit family, whereby these hotspot mutations reduced the rate of GTP hydrolysis of the active GTP-bound Gα subunit, resulting in constitutive signaling activity, human tumors carrying mutations that activate Gα12 have yet to be reported to date [25–27]. In fact, in head and neck cancers, Gα12 mutation frequency has been largely found to be low (1.3%). It is possible that mutations in GPCRs coupled to G12 family that are frequently found in other cancers provide an oncogenic advantage, hence additional gain-of-function mutations in these G-proteins may not be frequently observed [25]. Following our observation that high levels of Gα12 are predominantly detected in OSCC and not in NOM tissues, it is possible that Gα12 may acquire an oncogenic transforming ability in the overexpressed form, compared to other heterotrimeric Gα proteins which have to attain the constitutively GTP-bound form to induce transformation.

It is noteworthy that Gα12 and its G12 subfamily counterpart, Gα13, share 67% amino acid identity [6], and both appear to interact with the same receptors and downstream effectors in mediating cell signaling [28, 29]. In this study we determined that while Gα13 levels remained unchanged, Gα12 levels were observed to be largely overexpressed in our OSCC tissue cohort, indicating the importance of Gα12 in OSCC tumorgenesis and progression. In this context, studies have suggested that the functions of Gα12 and Gα13 may not completely overlap, whereby Gα12 has been demonstrated to be more potent in inducing oncogenic transformation including cell shape changes and migration [15, 30], while Gα13 was noted to be more active in inducing angiogenesis and apoptosis [12, 31].

Several studies have demonstrated that overexpression of Gα12 is associated with tumor invasiveness [16–18, 32]. In this regard, our IHC analysis of Gα12 on OSCC samples showed no association between Gα12 expression in the primary tumors and LN metastasis, but intriguingly high levels of this protein were found to be retained in the OSCC cells that had metastasized into the LN. More recently, during the preparation of this manuscript, Jian and colleagues [32] also found no association between Gα12 overexpression and LN status in OSCC patients, but showed that Gα12 overexpression significantly correlated with extracapsular spread.

We further examined the function of Gα12 in OSCC. Although both activation by the use of Gα12 GTPase defective mutants and overexpression of Gα12 have been demonstrated to have transformation properties [14–16], we focused here on the use of an activated Gα12 mutant as it may facilitate the analysis of its consequences and to minimize the variability that may be caused by relying on overexpression and likely activation by endogenously expressed Gα12-coupled receptors. Our *in vitro* data demonstrated that expression of constitutively activated-Gα12 increased OSCC cell migration and invasion. We also demonstrated that inhibition of Gα12 expression in OSCC cells by shRNA markedly inhibited OSCC cell migration and invasion. Perhaps it is not too surprising to find that deregulation of G12 promotes cell migration and invasion in cancer development, since physiologically, normal G12 proteins regulate cell polarity, cytoskeletal rearrangement and processes governing cell shape changes which are essential in controlling cell motility during embryonic development [8, 9]. In addition, they also regulate the migration of lymphocytes, neutrophils and vascular smooth muscle cells [33–36].

Signaling by G12 proteins are responsible for the Rho-dependent cytoskeletal changes required for cell migration [28, 34, 37, 38]. Activation of G12 signaling by PAR-1 and LPAR inducing cancer cell invasion have been shown to be mediated by Rho proteins in breast and prostate cancers [16, 17, 39]. Here, we investigated the G12-RhoA signaling axis in OSCC, whereby expression of RGS blocked G12 downstream signaling as indicated by the reduction in LPA- and thrombin-dependent RhoA activation. Blockade of G12 signaling by RGS resulted in the inhibition of OSCC cell migration *in vitro*. In support, RGS expression has been reported to block G12 signaling in triple-negative breast cancer cell lines, reduced SDF-1-dependent Rho activation, causing a reduction in invasion and migration [19]. Although we did not further examine the downstream G12 signaling mechanism in mediating cytoskeletal changes, it is possible that RhoA promotes changes in the cytoskeletal architecture through ROCK, and the remodeling of focal adhesion components through protein kinase C-related kinase to collectively cause cell-cell junction disruption [38]. Interestingly, G12 subfamily can couple to the CXCR4 chemokine receptor and drive tumor spread to specific organs in RhoA dependent manner [19]. Also, G12 can enhance cell motility and invasion independently of Rho proteins, such as by reducing cell-extracellular matrix adhesion through integrins [40], decreasing the rigidity of cell-cell contacts by reducing the stability of homophilic E-cadherin interactions [41], as well as by activating transcription factors that control the expression of metalloproteases such as matrix-metalloproteinase (MMP)-2 and MMP-9 [29]. In a recent study, Gα12 signaling was shown to mediate OSCC invasion by up-regulating proinflammatory cytokines [32], suggesting that Gα12 is a critical player in the inflammatory cytokines pathways during OSCC tumorigenesis. In support of these observation, we have recently reported that these proinflammatory cytokines (IL-6 and IL-8) were found to be elevated in the serum samples of OSCC patients using a novel antibody based screening device [42], suggesting that high Gα12 level may be causal for the induction of these cytokines.

More importantly, we further demonstrated that RGS mediated inhibition of G12 signaling significantly reduced the metastatic dissemination of OSCC cells from the primary tumor mass to the LNs irrespective of size, suggesting that G12 signaling does not impact the growth of the primary tumor but is likely to drive metastasis. In support of these observations, previous studies on breast cancer also showed that the blockade of G12 downstream signaling resulted in a notable impact on the metastatic spread that resulted in a significant increase in metastasis-free survival of mice [16]. As mentioned earlier, OSCC has a high potential to spread to the cervical LNs, therefore any strategies that may result in locoregional control of the disease will play an important role in improving patient survival. Thus, these findings suggest that interfering with the G12-Rho signaling axis and their key down-stream targets may provide previously unexplored options to halt OSCC metastasis.

Several studies have indicated that Gα12 may be an important promoter for cell growth in certain cell types, as this molecule in its wild-type and activated-form has been shown to be involved in the proliferation of fibroblasts [14, 15, 30, 43, 44]. In this study, both Gα12 expression and activation consistently showed no effect in OSCC cell proliferation in *in vitro* results, suggesting that Gα12 is not crucial in regulating OSCC cell growth and proliferation. In support of our *in vitro* findings, our *in vivo* data also suggested that the growth of primary tumors on the tongue were not affected, despite the reduction in LN metastasis when G12 signaling was inhibited by RGS. Our findings are in agreement with those reported by Kelly and colleagues [16, 17]. These differences seen in the role of G12 in controlling cell proliferation may be due to the intrinsic properties of the tumor and also the different cellular origin such as the mesenchymal (fibroblasts) versus epithelial cells, as Gα12 may exert specific effects through the different down-stream signaling pathways [37].

In conclusion, the overexpression of Gα12 in OSCC may be required for the amplification of G12 signaling to promote the transforming effects of this oncogene. Further, our data collectively support the functional significance of Gα12 in OSCC tumor metastasis that has also been reported in other human cancers [16, 17]. The presence of LN metastasis in OSCC represents the most important factor predicting a poor prognosis [45], and unfortunately there are still limited therapeutic options to prevent disease progression and OSCC spread. Many studies including ours have indicated that perturbing the G12 signaling may provide a reasonable approach to preventing metastasis in patients at risk.

## MATERIALS AND METHODS

### Tissue specimens

All clinical human tissues used in this study were collected with informed consent, and was approved by the ethical review board at the Faculty of Dentistry, University of Malaya (Ethical approval code: DFOP0703/0017). Frozen tissues comprising of 47 OSCC and 18 normal oral mucosa (NOM) tissues were used in this study. NOM specimens were obtained from the gingiva of individuals who did not have OSCC but had undergone wisdom tooth removal. In addition, the formalin-fixed paraffin-embedded (FFPE) tissue specimens used in this study include 42 OSCC specimens, 10 fibro-epithelial polyps (FEP), 13 NOM and 12 metastatic cervical LN specimens that are matched to primary tumors. The diagnoses of all tissue specimens used in this study were histopathologically confirmed by an oral pathologist (RBZ). The socio-demographic information of the patient cohort was obtained from the Malaysian Oral Cancer Database and Tissue Bank System (MOCDTBS) [46], and this is summarized in Table 2.

**Table 2:** Demographic distribution of 42 OSCC cases used in Gα12 IHC analysis

	Variables	OSCC	
		n	(%)
**Age (year)**	Mean ± SD: 61 ± 10		
**Gender**	Male	12	(28.6)
	Female	30	(71.4)
**Ethnic**	Malay	6	(14.3)
	Chinese	3	(7.1)
	Indian	32	(76.2)
	Others	1	(2.4)
**Primary site**	Cheek	25	(59.5)
	Gum	11	(26.2)
	Tongue	5	(11.9)
	Others	1	(2.4)
**Habit**	No habit	4	(9.5)
	Chew betel quid	22	(52.4)
	Smoking	4	(9.5)
	Drink alcohol	1	(2.4)
	More than 1 risk habit	12	(28.6)

### RNA extraction and cDNA synthesis

Total RNA was extracted from frozen tissues containing at least 70% tumor cells (OSCC specimens) or normal epithelial cells (for NOM) using the RNeasy Micro kit (Qiagen, Germany), according to the manufacturer's instructions. The quality and quantity of RNA extracted from these samples were evaluated using the Agilent 2100 Bioanalyzer (Agilent Technologies, USA). cDNA was synthesized from 2 μg of total RNA using the High Capacity cDNA Reverse Transcription kit (Applied Biosystems, CA, USA) in a total volume of 100 μl, as described previously [20].

### Quantitative PCR (qPCR)

qPCR was performed with standard SYBR Green protocol using the ABI PRISM® 7000 Sequence Detection System (Applied Biosystems, Germany), as described previously [20]. Briefly, reactions were carried out by performing a pre-incubation for 10 minutes at 95°C, followed by 40 amplification cycles at 95°C for 15 seconds and 60°C for 1 minute. The primers used were as follow:

Gα12 sense 5′ATAAGTCAGATTGTTAACTCCAAGATTGA3′

Gα12 antisense 5′AGCCAGACCCTCCCAATGTT3′

Gα13 sense 5′GGGCAGGACTTCGACCAGCG3′

Gα13 antisense 5′CCATGGGGGCCCGGGTATCA3′

GAPDH sense 5′GAAGGTGAAGGTCGGAGTC3′

GAPDH antisense 5′GAAGATGGTGATGGGATTTC3′

Relative quantification was performed by comparative Ct method to normalize the expression of Gα12 and Gα13 to the housekeeping gene GAPDH. Relative expression of target genes in OSCC were determined against Ct values of the NOM tissues.

### Immunohistochemistry (IHC)

The expression of Gα12 was examined by IHC using the Dakocytomation Envision+ Dual Link System-HRP (DAB+) kit (Dako, Glostrup, Denmark). IHC was performed on the FFPE primary OSCC tissues and 12 available matching lymph node tissues as described previously [47]. The Gα12 polyclonal antibody (SC-409; Santa Cruz Biotechnology, CA, USA) was used as the primary antibody at a dilution of 1:75. Immunoreactivity of epithelial cells (cancer and normal) was scored based on a 4-point intensity scoring system: 0 = negative expression; 1 = weak positive; 2 = moderate positive; 3 = strong positive as described previously [16, 17]. All IHC analysis was evaluated independently by a pathologist (RBZ). The receiver operating characteristic (ROC) curve was used to identify the best cut-off points in scoring the expression of Gα12 for specificity and sensitivity, whereby scores of 1 or 2 would be grouped as low expression, while 3 or 4 would be considered as high expression.

### Cell culture

Asian OSCC cell lines (ORL48, ORL115, ORL150, ORL153, ORL174, ORL188, ORL207, ORL214) used in this study were previously established in our laboratory as described [47]. Prior to use, all lines underwent authentication using the AmpF/STR^®^ Identifier^®^ PCR Amplification kit (Applied Biosystems, USA). All OSCC cell lines were cultured in Dulbecco's Modified Eagle's Medium/Nutrient mixture F-12 HAM's medium (DMEM/F-12; Hyclone, Utah, USA) supplemented with 500 ng/ml hydrocortisone (Sigma-Aldrich, MO, USA), and 10% fetal bovine serum (FBS; Gibco, Auckland, NZ). Normal oral keratinocytes primary cultures (ORL218 and ORL232) established in our laboratory from healthy gingival biopsies, were maintained in Keratinocyte Serum-Free Medium (KSFM; Gibco, Auckland, NZ) supplemented with 25 μg/ml bovine pituitary extract, 0.4 ng/ml epidermal growth factor, and 0.09 mM CaCl_2_. Mouse NIH3T3 and packaging cell lines HEK-293T, GP-293 (Clontech, CA, USA), were grown and maintained in DMEM high glucose medium (Gibco, Auckland, NZ) containing 10% FBS.

### Overexpression of activated Gα12 in OSCC

The Gα12QL plasmid was a kind gift from Dr. Patrick Casey (Duke University Medical Centre). GP-293 packaging cells were grown in a 60 mm dish until 70% confluence (~24 hours) prior to transfection. Retroviral stock was prepared by co-transfecting 5 μg of pLXRN (vector control [VC]) or Gα12QL with 5 μg of pVSVG (envelope) into the GP-293 packaging cells using Lipofectamine 2000 (Invitrogen, CA, USA), according to the manufacturer's instructions. The supernatants containing the retrovirus were harvested after 48 hours and filtered through 0.45 μm PVDF syringe filter. ORL150 cells expressing low levels of Gα12 were seeded at 1×10^5^ cells in a 60 mm dish 18 hours prior to retroviral infection. Then, filtered viral supernatant was added onto the ORL150 cells with 10 μg/ml polybrene (Sigma-Aldrich, MO, USA). Successfully transduced cells were selected with 75 μg/ml G418 (Sigma-Aldrich, MO, USA).

### Inhibition of G12 signaling by RGS

The green fluorescence protein (GFP)-fused to the regulator of G-protein signaling (RGS) domain of PDZ-RhoGEF has been previously described by Basile and colleagues [48]. HEK-293T packaging cells were grown in a 6-well plate until 70% confluence (~24 hours) prior to transfection. Lentiviral stock was prepared by co-transfecting RGS expression vector with pSPAX2 (packaging) and pVSVG (envelope) vectors in the ratio of 3:1:1 into HEK-293T with Turbofect (Fermentas, MD, USA) and thereafter, the cells were culture for an additional 48 hours. The supernatants containing lentiviruses were harvested and filtered through 0.45μm PVDF syringe filter. ORL48 cells expressing high levels of Gα12 were seeded at 2×10^5^ cell density in 6-well plates 18 hours prior to lentiviral infection. ORL48 cells were incubated with filtered lentiviral supernatant for 48 hours with 10 μg/ml polybrene (Sigma-Aldrich, MO, USA). Virus titre efficiency was evaluated under a fluorescence microscope (Olympus, Japan) to determine the percentage of cells that express GFP. These cells were further sorted by FACS (Beckman Coulter, Fullerton, CA) to harvest only GFP-positive cells and these were further expanded in normal growth medium for down-stream experiments.

### Gα12 knockdown by RNA interference

The pGIPz vectors containing Gα12 shRNA target sequences:

shGα12 (388311): 5′GGCAAGTCCACGTTCCTCA and shGα12 (388313): 5′GAGACCATCGTCAACAACA3′ were purchased from Open Biosystems (Thermo Fisher Scientific, AL, USA), and used to knockdown the protein expression of Gα12 in ORL48 cells, essentially following the lentivirus production method as stated above. Viral titration efficiency was evaluated under a fluorescence microscope (Olympus, Japan) to determine the percentage of cells expressing GFP. Transduced cells were selected using 7 μg/ml puromycin (Sigma-Aldrich, MO, USA) and maintained thereafter in medium containing puromycin.

### Western blotting

Cells were lysed on ice in lysis buffer (0.5% NaDOC, 0.1% SDS, 25mM HEPES pH 7.5, 0.3 M NaCl, 1.5 mM MgCl_2_, 0.2 mM EDTA, 1% Triton X-100, 0.5 mM DTT, 20 mM β-glycerophosphate, 0.1mM Na_3_VO_4_) supplemented with 1x HALT protease inhibitor cocktail (Pierce Biotechnology, IL, USA). Cell lysates were collected after centrifugation at 14000 rpm for 5 minutes at 4°C. The concentration of total protein in the cell lysate was determined using Bradford protein assay (Pierce Biotechnology, IL, USA). 50 μg of total protein was subjected to 12% SDS-polyacrylamide gel electrophoresis and transferred onto Immobilon-P membrane (Millipore, MA, USA). Blots were blocked with 5% skimmed milk (TBS with 0.1% Tween-20) for 1 hour and further incubated with the following primary antibodies: anti-Gα12 (sc-409; Santa Cruz Biotechnology, CA, USA), anti-Gα13 (sc-410; Santa Cruz Biotechnology, CA, USA) and anti-α-tubulin (T9026; Sigma-Aldrich, MO, USA), at a 1:1000 dilution for 1 hour. Blots were washed (x3 for 5 minutes each) in TBS buffer containing 0.1% Tween-20 (TBS-T) and then probed with respective secondary antibodies conjugated with horseradish peroxidase (dilution 1:10,000; SouthernBiotech, AL, USA) for 1 hour. After washing (TBS-T), detection was performed via enhanced chemiluminescence method, using the ChemiImager™ Imaging System (Alpha Innotech, USA). Quantification of protein bands was performed using the ImageJ software (http://rsbweb.nih.gov/ij/). The relative intensity of target proteins was calibrated against α-tubulin and then compared to the control for each experiment.

### RhoA pull-down assay

RhoA activity in cultured cells was assessed as described by Chikumi and colleagues [49]. Briefly, cells were grown to 70% confluence in 100 mm dishes, serum-starved for 18 hours and then stimulated with 1U/ml thrombin (Sigma, MO, USA) or 5 uM lysophosphatidic acid (LPA) (Sigma-Aldrich, MO, USA) for 30 seconds to 10 minutes. Cells were lysed on ice with 600 μl of cold lysis buffer (detailed above). Lysates were then collected and centrifuged at 14000 rpm for 5 minutes at 4°C. From the resulting supernatants, 100 μl was used for total RhoA protein levels, while the remaining 500 μl for each sample were incubated with glutathione-Sepharose 4B beads (GE Healthcare, Sweden) bound to glutathione-S-transferase (GST)-rhotekin-RhoA binding domain for 30 minutes at 4°C on a rotator. The beads were collected by centrifugation at 9000 rpm for 1 minute at 4°C and washed (x3) with cold lysis buffer. The associated GTP-bound RhoA was released with protein loading buffer and denatured by heating for SDS-polyacrylamide gel electrophoresis followed by western blotting. Blots were incubated using anti-RhoA (sc-418; Santa Cruz Biotechnology, CA, USA) at 1:1000 dilution for 1 hour and further processed according to western blotting conditions mentioned above.

### Cell proliferation

Cells were seeded at 2×10^4^ density in 6-well plates, and cultured for up to 7 days. Cell counts were performed every 24 hours using the CASY cell counter (Innovatis AG, Reutlingen, Germany), according to manufacturer's instructions. Each experiment was performed in triplicates, and mean cell counts were compared to the respective vector controls across different time intervals.

### Scratch assay

Scratch assays were performed as previously described [50]. In brief, cells were seeded at 4×10^5^ cells/ml and cultured for 48 hours to form a confluent monolayer. Cells were then treated with 10 μg/ml mitomycin C for 2 hours to inhibit cell proliferation. A scratch was made through the monolayer with a P200 pipette tip by applying constant pressure to create an open wound. Cells were rinsed with PBS and cultured in DMEM/F-12 complete medium and open wound areas were microscopically recorded between 0 to 18 hours. Images of open wounds were then analyzed using the TScratch analysis software [51].

### Transwell migration and matrigel invasion

Migration assays were performed using 24-well Transwell chambers with polyethylene terephthalate (PET) membrane of 8 μm pore size (BD Biosciences, MA, USA). Cells were serum-starved for 18 hours and detached with Trypsin-EDTA (Gibco, Auckland, NZ). Then, 1×10^5^ cells were suspended in serum-free medium and added to the upper chambers of the Transwell inserts and 3T3 cell-conditioned medium or medium containing 1U/ml thrombin was used as chemoattractant in the lower chamber. After 48 hours of incubation at 37°C, cells in the upper surface of the membranes were removed with a cotton swab. The membranes were fixed with 4% formaldehyde for 15 minutes and stained with 0.2% crystal violet for 10 minutes, and further rinsed with water. The membranes were detached from the inserts and mounted onto glass slides using DPX mountant. Stained cells were counted in 4 randomly chosen microscopic fields per insert at 200x magnification and average value of 3 biological replicates was obtained.

Cell invasion was assayed with Biocoat Matrigel 24-well invasion chamber (BD Biosciences, MA, USA) according to the method described for the Transwell migration assay, with the exception that inserts were pre-coated with matrigel basement matrix (BD Biosciences, MA, USA).

### Animal studies

All animal studies were carried out according to National Institutes of Health-approved protocols in compliance with the guide for the care and use of laboratory animals. Female NOD/SCID mice (NCI, Frederick, MD, USA) at 4–6 weeks of age were used in this study. Mice were housed in appropriate sterile filter-capped cages, and fed and watered *ad libitum*. The orthotopic tongue model was established based on a previous study by Patel and colleagues [22]. Briefly, 1×10^5^ ORL48/GFP and ORL48/RGS cells previously sorted for GFP by FACS were injected into the posterior tongue. Mice were evaluated every other day for general behavioral abnormalities, signs of illness or discomfort. Body weights of the animals were recorded and tumor measurements were given visually by the same operator for the duration of the study. Mice were euthanized on day 20 and the neck area of each mouse was carefully dissected under a Discovery V12 Stereo microscope (Zeiss, Thornwood NY, USA) to retrieve 4 to 5 cervical lymph nodes. Next, the tongues for each mouse in each group were resected. The resected tissues were fixed in formalin overnight and then transferred to 70% alcohol and processed for paraffin embedding for histopathological evaluation by a pathologist (AAM). H&E-stained slides from primary tumors were acquired with an Aperio CS Scanscope (Aperio, Vista, CA, USA) at 40x magnification. Tumor volume was determined using the formula: LW^2^/2; where L and W represent length and the width of the tumor respectively.

### Statistical analyses

Statistical analyses were performed using SPSS 16 (SPSS Inc., Chicago, IL, USA) software. Gα12 mRNA levels in OSCC and NOM tissues were compared using the Mann-Whitney U test. The receiver operating characteristic (ROC) curve was used to determine the cut-off point for the IHC scoring of Gα12 expression in differentiating the tumors from the NOM tissues. Fisher-exact test was used to study the association between Gα12 protein expression in OSCC and various clinicopathological parameters. Independent t-test was used to analyze the differences in *in vitro* cell proliferation, invasion and migration, as well as in *in vivo* experiments comparing the tumor volume and LN involvement between the two groups of animals.

## SUPPLEMENTARY FIGURES


