# Supplementary material for: Heterotrimeric G-protein alpha-12 (Gα12) subunit promotes oral cancer metastasis
Source: Oncotarget. 2014 Nov 12;5(20):9626–40. doi: 10.18632/oncotarget.2437 (PMC4259425; doi:10.18632/oncotarget.2437)
Supplement: Supplementary file 1 [file oncotarget-05-9626-s001.pdf]

SUPPLEMENTARY FIGURES

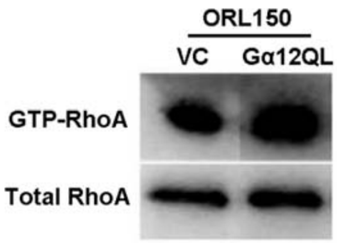

Supplementary Figure S1: Expression of Gα12QL in ORL150 induces RhoA activation.

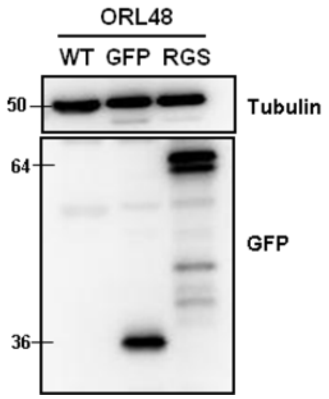

Supplementary Figure S2: Expression of GFP and the GFP-RGS domain of PDZ-RhoGEF in ORL48 was determined using anti-GFP antibody. The presence of band at the size of 75kDa indicates the expression of GFP-RGS domain of PDZ-RhoGEF, whereas the control vector only shows expression of GFP at 35kDa.

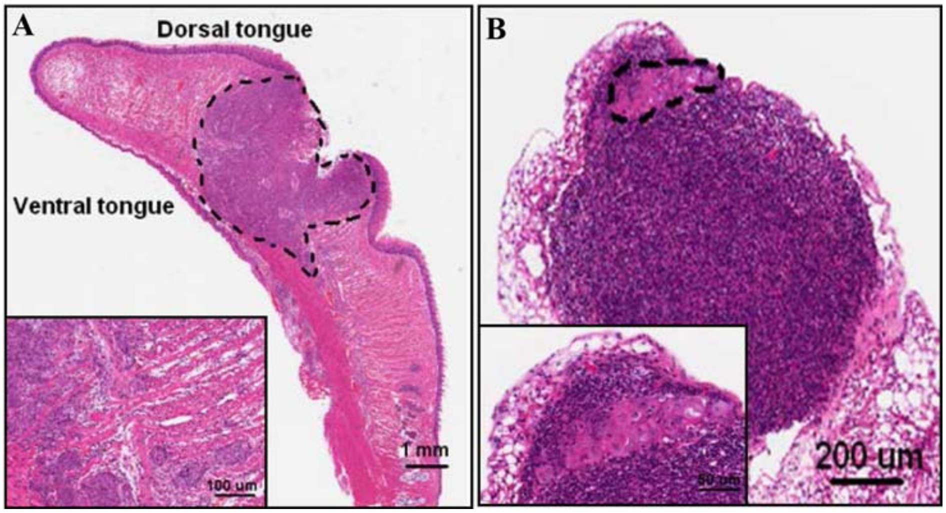

Supplementary Figure S3: Histology evaluation of ORL48 orthotopic tongue xenograft. **(A)** H&E section showing the growth of the primary tumor on the tongue. Inset is the higher magnification of the tumoral area showing ORL48 cells formed moderately differentiated tumor with intramuscular invasion. **(B)** H&E section of the cervical LN indicated the presence of OSCC metastasis. Inset showed the higher magnification of the metastatic LN area.
